# Investigating causal relationships between exposome and human longevity: a Mendelian randomization analysis

**DOI:** 10.1186/s12916-021-02030-4

**Published:** 2021-07-20

**Authors:** Shu-Yi Huang, Yu-Xiang Yang, Shi-Dong Chen, Hong-Qi Li, Xue-Qing Zhang, Kevin Kuo, Lan Tan, Lei Feng, Qiang Dong, Can Zhang, Jin-Tai Yu

**Affiliations:** 1 Department of Neurology and Institute of Neurology, Huashan Hospital, State Key Laboratory of Medical Neurobiology and MOE Frontiers Center for Brain Science, Shanghai Medical College, Fudan University, 12th Wulumuqi Zhong Road, Shanghai, 200040 China; 2 Department of Neurology, Qingdao Municipal Hospital, Qingdao University, Qingdao, China; 3 Department of Psychological Medicine, Yong Loo Lin School of Medicine, National University of Singapore, Singapore, Singapore; 4 Genetics and Aging Research Unit, McCance Center for Brain Health, Mass General Institute for Neurodegenerative Diseases (MIND), Department of Neurology, Massachusetts General Hospital, Harvard Medical School, Charlestown, MA USA

**Keywords:** Longevity, Mendelian randomization, Exposome

## Abstract

**Background:**

Environmental factors are associated with human longevity, but their specificity and causality remain mostly unclear. By integrating the innovative “exposome” concept developed in the field of environmental epidemiology, this study aims to determine the components of exposome causally linked to longevity using Mendelian randomization (MR) approach.

**Methods:**

A total of 4587 environmental exposures extracting from 361,194 individuals from the UK biobank, in exogenous and endogenous domains of exposome were assessed. We examined the relationship between each environmental factor and two longevity outcomes (i.e., surviving to the 90th or 99th percentile age) from various cohorts of European ancestry. Significant results after false discovery rates correction underwent validation using an independent exposure dataset.

**Results:**

Out of all the environmental exposures, eight age-related diseases and pathological conditions were causally associated with lower odds of longevity, including coronary atherosclerosis (odds ratio = 0.77, 95% confidence interval [0.70, 0.84], P = 4.2 × 10^−8^), ischemic heart disease (0.66, [0.51, 0.87], P = 0.0029), angina (0.73, [0.65, 0.83], P = 5.4 × 10^−7^), Alzheimer’s disease (0.80, [0.72, 0.89], P = 3.0 × 10^−5^), hypertension (0.70, [0.64, 0.77], P = 4.5 × 10^−14^), type 2 diabetes (0.88 [0.80, 0.96], P = 0.004), high cholesterol (0.81, [0.72, 0.91], P = 0.0003), and venous thromboembolism (0.92, [0.87, 0.97], P = 0.0028). After adjusting for genetic correlation between different types of blood lipids, higher levels of low-density lipoprotein cholesterol (0.72 [0.64, 0.80], P = 2.3 × 10^−9^) was associated with lower odds of longevity, while high-density lipoprotein cholesterol (1.36 [1.13, 1.62], P = 0.001) showed the opposite. Genetically predicted sitting/standing height was unrelated to longevity, while higher comparative height size at 10 was negatively associated with longevity. Greater body fat, especially the trunk fat mass, and never eat sugar or foods/drinks containing sugar were adversely associated with longevity, while education attainment showed the opposite.

**Conclusions:**

The present study supports that some age-related diseases as well as education are causally related to longevity and highlights several new targets for achieving longevity, including management of venous thromboembolism, appropriate intake of sugar, and control of body fat. Our results warrant further studies to elucidate the underlying mechanisms of these reported causal associations.

**Supplementary Information:**

The online version contains supplementary material available at 10.1186/s12916-021-02030-4.

## Background

Longevity is defined as the length or duration of life or viability, typically refer to the age of death or survival beyond of 90–100 years or older [1]. It is a heterogenous trait that is susceptible to genetic and environmental factors. Previous genome-wide association studies (GWASs) have revealed genetic loci associated with human longevity or parental lifespan [2, 3], while environmental factors, including socio-economic status, smoking, gender, and lifestyle, are considered determinants [1]. Observational studies have also featured the associations on various risk factors, where the predicted longevity could be significantly reduced by cardiovascular disease (CVD), diabetes, hypertension, and tobacco smoking [4, 5]. However, due to the vulnerability to reverse causation and confounding bias, most of the epidemiological studies are insufficient to draw a definite conclusion on causality.

Mendelian randomization (MR) is an analytical approach that can overcome such limitations by using genetic variants as instrumental variables (IVs) to evaluate the causal effect of exposure on the outcome. Since genotypes are randomly allocated from parents to offspring [6], MR method is less likely to be affected by reverse causality and measurement errors in the absence of pleiotropy, making causal inference more feasible compared to conventional study designs. Although several MR analyses have demonstrated a subset of environmental factors that were causally associated with longevity [7–9], the exploration of causal exposures is still in a relatively primitive stage. However, by applying the “exposome” concept proposed in the field of environmental epidemiology, we are able, for the first time, to investigate the totality of environmental exposures that affect an individual from conception until death [10]. Using the MR approach, our study aims to construct the potential components of exposome that causally linked to longevity.

## Methods

### Exposure data

**Fig. 1:**
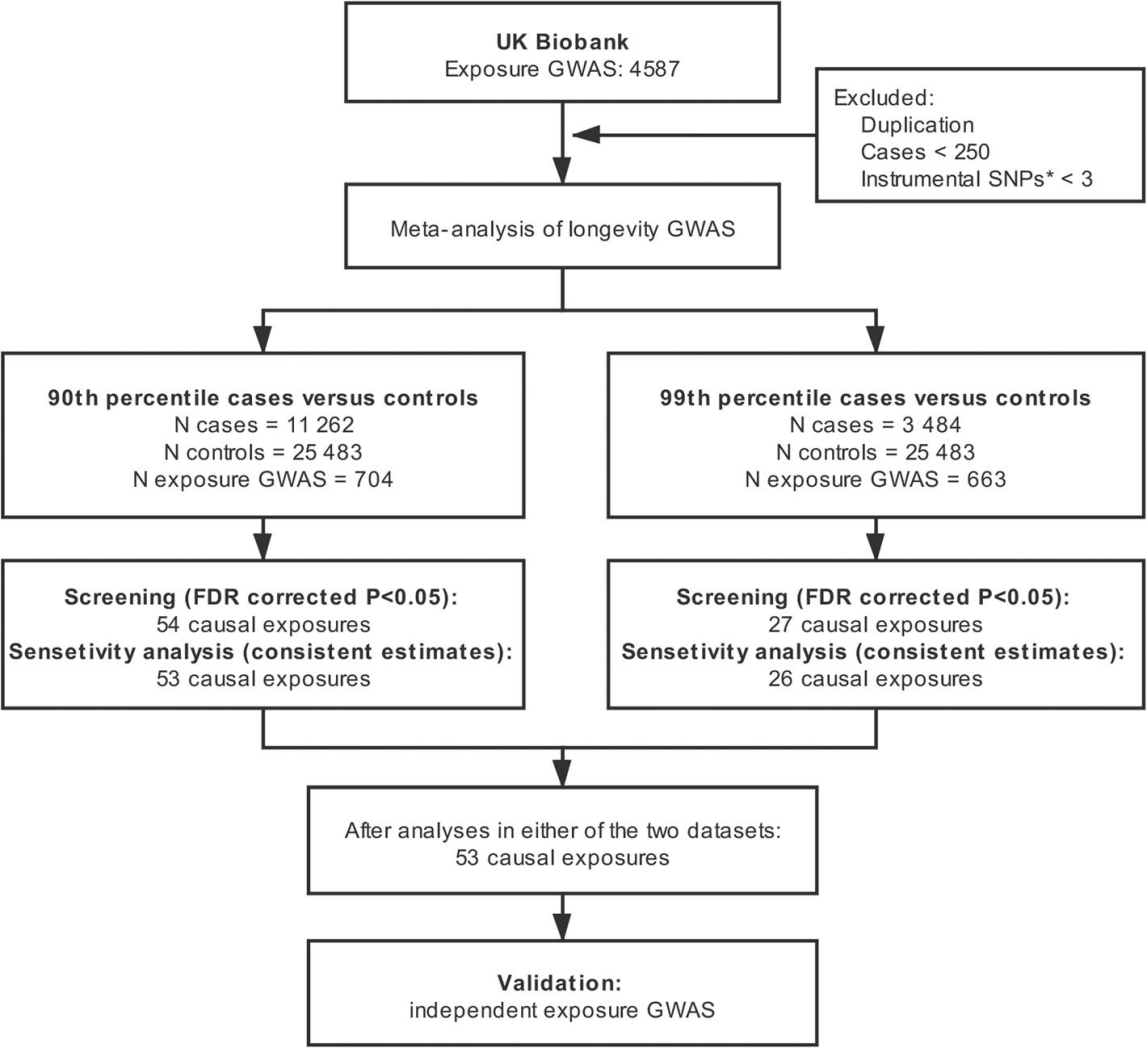
A flow diagram of the study design and analysis process. FDR, false discovery rates; GWAS, genome-wide association study; N, number or sample size; SNPs, single nucleotide polymorphisms

UK Biobank (UKB) is a large-scale and long-term biobank with information on both genetics and broad environmental exposures collected over 10 years (www.ukbiobank.ac.uk). Over 500,000 individuals aged 40–69 years were recruited from across the UK between 2006 and 2010. The exposome data used in our MR analysis were originally from the UK Biobank. GWAS summary statistics of 4587 environmental exposures were obtained from the Neale Lab (http://www.nealelab.is/uk-biobank), based on 361,194 participants [11]. Categorical exposures with cases < 250 and duplicated exposures were excluded [12]. Exposures with less than three independent single nucleotide polymorphisms (SNPs) at P < 5 × 10^−8^ were also excluded (Fig. 1). Finally, a total of 704 exposures were included in primary analysis, and 663 exposures were included in secondary analysis. We classified all these available exposures into three main domains: endogenous, exogenous individual and exogenous macro-level [13]. Exposures in each domain were then classified into different categories, mainly according to information in UKB.

### Outcome data

We used two summary statistics from the largest meta-analysis of human longevity GWAS of European ancestry [3]. Longevity was defined as two dichotomous phenotypes [3]. Cases were individuals who lived beyond the 90th (N = 11,262) or 99th (N = 3484) percentile. Controls (N = 25,483) were individuals who died at or before the age at the 60th percentile or whose age at the last follow-up visit was at or before the 60th percentile age. To mitigate the heterogeneity, the cohort-specific life tables for the country, sex, and birth, are used to identify the age threshold for cases and controls in the original GWAS [3]. Hence, the number of selected cases and controls is independent of the study population used. The 90th percentile longevity data was used in the primary analysis because of the larger sample size, while 99th percentile data was used as secondary analysis. The mean age of 90th percentile cases was 97 years, ranging from 87 to 122. The mean age of 99th percentile cases was 101 years, ranging from 90 to 122. The mean age of the control group was 55 years, ranging from 0 to 88. All participants provided written informed consent in original GWAS [3].

### Two-sample MR design

We inferred causal relationships between each environmental exposure and longevity using two-sample MR, in which the selections of IVs are based on GWAS summary statistics generated from different, non-overlapping samples. To obtain unbiased estimates of the causal effects, MR analysis rests on three assumptions [6]: (i) the genetic variants are associated with the exposure, (ii) the genetic variants are independent of confounders of the risk factor–outcome association, and (iii) the genetic variants influence the outcome only through the exposure.

### Selection of instrumental variables

For each exposure, single nucleotide polymorphisms (SNPs) associated at P-value < 5 × 10^−8^ with a minor allele frequency greater than 0.01 were considered potential instruments. We used MR-Base (http://www.mrbase.org) to select independent SNPs at a linkage disequilibrium threshold of r^2^ < 0.001, and retained SNPs with the strongest effect on the associated trait. For palindromic SNPs, we aligned strands using allele frequency and discarded palindromic SNP(s) that had minor allele frequency above 0.42. Then, exposure–outcome datasets were harmonized. We have considered the palindromic SNPs and checked original datasets to avoid reverse effects.

We computed the F-statistic of each exposure to judge the strength of IVs. The bias from weak instruments depends on the strength of the instrument through the F-statistic, which is related to the proportion of variance in the phenotype explained by IVs (R^2^), sample size (n) and number of instruments (k) by the formula F = $\left(\frac{n-k-1}{k}\right)\left(\frac{R^2}{1-{R}^2}\right)$ [14]. Typically, a strong instrument was defined as an F-statistic > 10 [14]. We estimated the statistical power with a false positive rate α = 0.05 using R code provided by Burgess S [15]. Details of the genetic instruments were presented in Additional file 1: Table S1.

### Statistical analysis

We used the inverse variance weighted (IVW) method as our principal MR analytical approach. This method will return an unbiased estimate in the absence of horizontal pleiotropy or when horizontal pleiotropy is balanced. Results are presented as odds ratio (OR) per standard deviation (SD) increase in genetically determined metabolites on AD for the outcome was dichotomous. For the Neal lab GWAS data using a linear model (rather than a logistic model) when analyzing case-control traits, thus, we applied a transformation according to the manual of BOLT_LMM (https://alkesgroup.broadinstitute.org/BOLT-LMM/downloads/BOLT-LMM_v2.3.4_manual.pdf) in order to convert SNP effect estimates (“betas”) on the quantitative scale to traditional ORs. This approximate transformation is log OR = β/(μ × (1 − μ)), where μ = case fraction. Standard errors of SNP effect size estimates are also be divided by (μ × (1 − μ)) when applying that transformation to obtain log ORs.

Sensitivity analyses were conducted using weighted median [16], MR-Egger regression [17], and Mendelian randomization pleiotropy residual sum and outlier (MR-PRESSO) [18]. These methods hold different assumptions at the costs of reduced statistical power. The weighted median allows for 50% of the IVs to be invalid or present pleiotropy [16]. MR-Egger regression allows > 50% of the variants to be invalid [17]. Heterogeneity in the IVW estimates was examined by Cochran’s Q test. Furthermore, MR-Egger intercept and MR-PRESSO global test were used to check for the presence of pleiotropy. In the case of horizontal pleiotropy, MR-PRESSO outlier test compares the observed and expected distributions of the tested variants to identify outlier variants. If significant outliers (P < 0.05) are detected, they were removed from the analysis to return an unbiased causal estimate [18].

To correct for multiple comparisons, we applied false discovery rates (FDR) correction in IVW. An FDR corrected P-value < 0.05 was considered significant, and an unadjusted P-value < 0.05 was considered the evidence of a suggestive association. The significant traits with consistent point estimates across sensitivity analyses and IVW estimates were selected in the screening phase as the most robust causal exposures. Analyses were conducted using R version 3.6.3, with the MR analysis performed using the “TwoSampleMR” package version 0.5.2 [19].

### Validation

For those identified significant exposures, we used non-UKB GWAS to validate our MR results. A total of 20 independent GWAS data were publicly available as part of the MRbase package [19]. If more than one GWAS were available for a given trait, an optimal one was selected based on large sample size, sufficient available SNPs, both sexes, and European or mixed descent. Details of independent exposure GWAS were presented in Additional file 1: Table S2. For each trait in the validation, IVs were constructed starting from all SNPs with P < 5 × 10^−8^. In validation analysis, the IVs of low-density lipoprotein cholesterol (LDL-C), high-density lipoprotein cholesterol (HDL-C), and triglycerides are partly overlapped [20]. Thus, we used multivariable MR to adjust for the genetic correlation [21]. Validation analyses were not conducted for those significant exposures without eligible data.

## Results

### Screening results

**Fig. 2:**
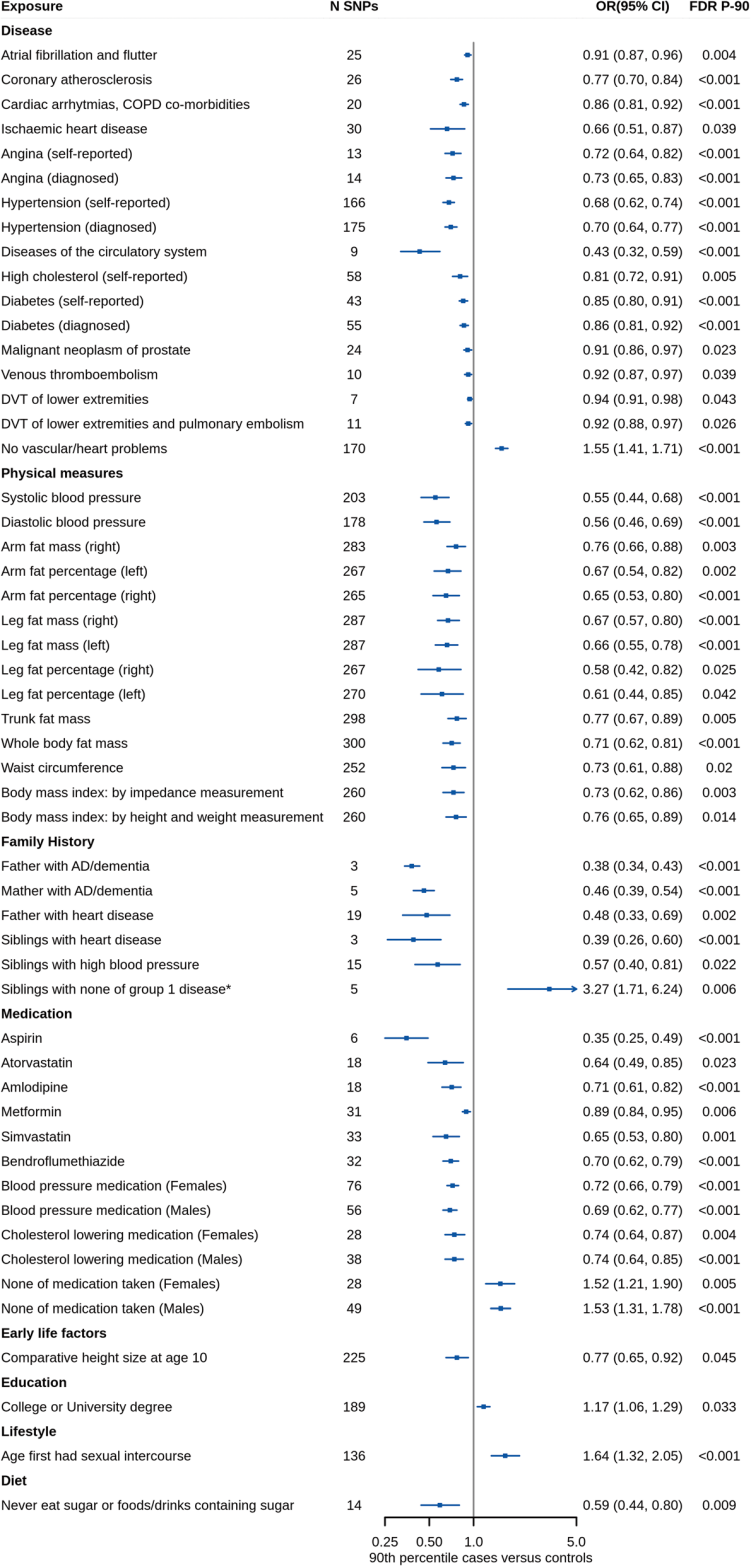
Mendelian randomization estimates for association between genetically predicted exposures and longevity in primary analysis. The estimates present here were calculated by the IVW method. *Group 1: heart disease, stroke, high blood pressure, chronic bronchitis/emphysema, and Alzheimer’s disease/dementia, diabetes. AD, Alzheimer’s disease; CI, confidence interval; COPD, chronic obstructive pulmonary diseases; DVT, deep venous thrombosis; FDR, false discovery rates; N, number or sample size; OR, odds ratio; SNPs, single nucleotide polymorphisms

**Fig. 3:**
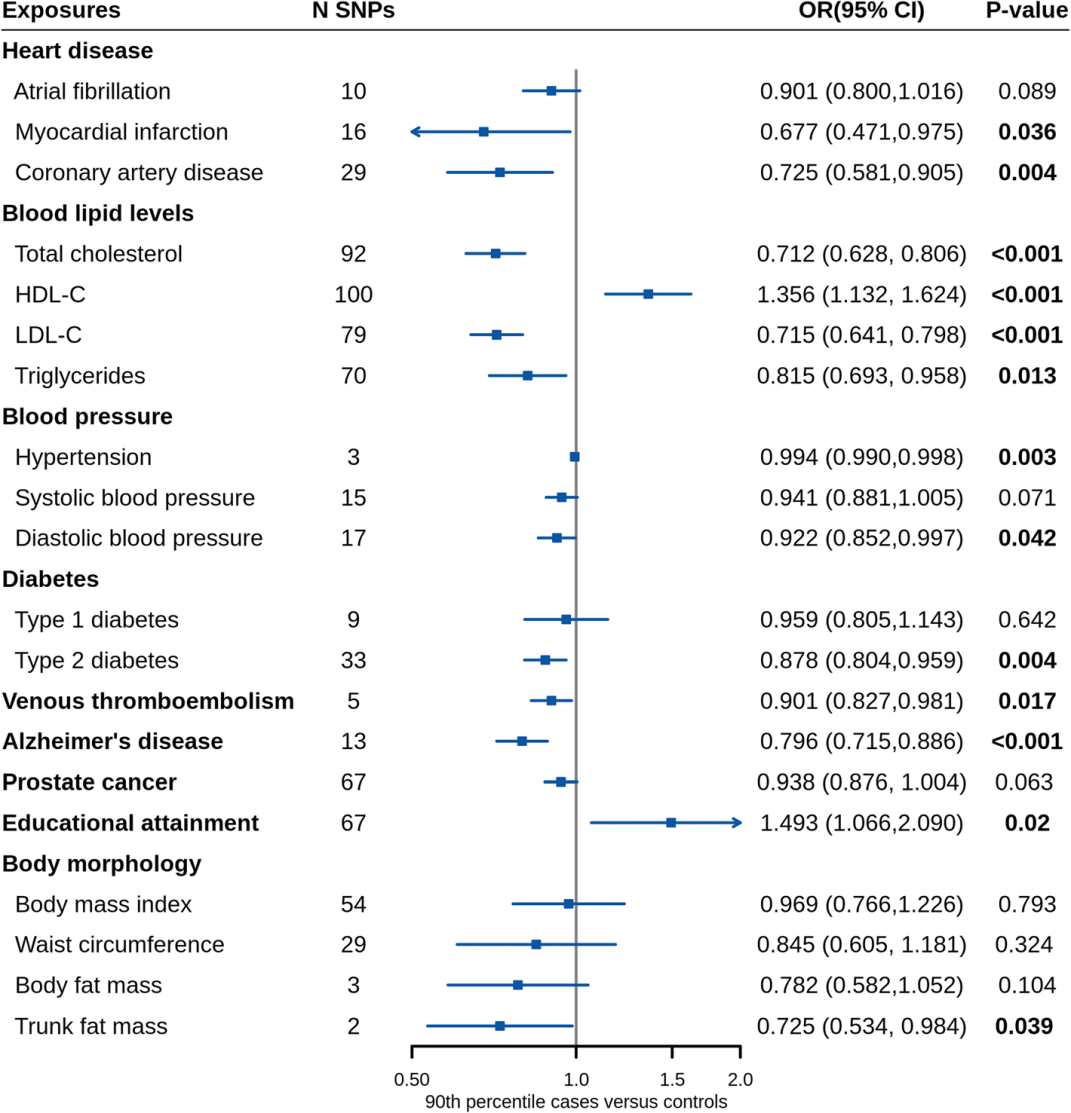
Mendelian randomization results of validation using independent exposure GWAS. The estimates present here were calculated by the IVW method. CI, confidence interval; HDL-C, high-density lipoprotein cholesterol; LDL-C, low-density lipoprotein cholesterol; N, number or sample size; OR, odds ratio; SNPs, single nucleotide polymorphisms

Of all analyzed exposures, 110 exposures and 73 exposures showed associations with longevity at P < 0.05 in the primary analysis and secondary analysis, respectively. We found that 53 exposures showed significant associations with either or both 90th and 99th percentile longevity after FDR correction and sensitivity analysis (Fig. 1). Of the 53 screening exposures, sensitivity analysis showed consistent point estimates with IVW in primary stage (Table 1). These exposures were classified into eight categories, including disease, physical measures, family history, medication, early life factors, education, lifestyle, and diet (Fig. 2, and Additional file 1: Table S3-S5). The list of the SNPs used as IVs for each screening exposure was presented in Additional file 1: Table S6. MR analyses were repeated using non-UKB exposure datasets (Fig. 3, and Additional file 1: Table S7-S9). A list of overview results in the present study is showed in Additional file 1: Table S10. All the significant and suggestive causal exposures from two longevity datasets are presented in Additional file 1: Table S11-S12. MR results of all traits are presented in Additional file 2: Table S13-S14.

**Table 1:** Sensitivity analysis results for the metabolites identified in primary analysis

Exposome components	Sample size	Cases	N SNPs	MR-Egger		Weighted median		MR-PRESSO	
				OR (95% CI)	P-value	OR (95% CI)	P-value	OR (95% CI)	P-value
**Disease**									
Atrial fibrillation and flutter	361194	6356	25	0.92 (0.82, 1.03)	0.148	0.93 (0.87, 1.00)	0.062	0.91 (0.49, 1.7)	**1.53 × 10**^**−04**^
Coronary atherosclerosis	361194	14334	26	0.64 (0.5, 0.82)	**3.69 × 10**^**−04**^	0.73 (0.65, 0.83)	**7.61 × 10**^**−07**^	0.77 (0.43, 1.35)	**1.07 × 10**^**−05**^
Cardiac arrhythmias, COPD co-morbidities	361194	8801	20	0.93 (0.79, 1.09)	0.349	0.9 (0.81, 0.99)	**0.032**	0.86 (0.47, 1.57)	**6.77 × 10**^**−05**^
Ischemic heart disease	361194	20857	30	0.53 (0.28, 1.03)	0.061	0.70 (0.60, 0.80)	**8.07 × 10**^**−07**^	0.66 (0.11, 3.99)	**0.006**
Angina (self-reported)	361141	11370	13	0.56 (0.37, 0.84)	**0.005**	0.75 (0.64, 0.89)	**6.88 × 10**^**−04**^	0.72 (0.24, 2.14)	**2.19 × 10**^**−04**^
Angina (diagnosed)	360420	11372	14	0.54 (0.36, 0.81)	**0.003**	0.75 (0.63, 0.89)	**0.001**	0.73 (0.27, 2)	**1.58 × 10**^**−04**^
Hypertension (self-reported)	361141	93560	166	0.77 (0.58, 1.03)	0.076	0.69 (0.61, 0.78)	**4.23 × 10**^**−09**^	0.68 (0.64, 0.72)	**3.30 × 10**^**−14**^
Hypertension (diagnosed)	360420	97139	175	0.74 (0.56, 0.98)	**0.037**	0.7 (0.62, 0.78)	**1.93 × 10**^**−09**^	0.7 (0.66, 0.75)	**2.41 × 10**^**−12**^
Diseases of the circulatory system	361194	60504	9	0.24 (0.09, 0.65)	**0.005**	0.42 (0.28, 0.63)	**3.48 × 10**^**−05**^	0.43 (0.23, 0.8)	**5.52 × 10**^**−04**^
High cholesterol (self-reported)	361141	43957	58	0.73 (0.57, 0.93)	**0.010**	0.83 (0.73, 0.94)	**0.005**	0.81 (0.59, 1.1)	**5.80 × 10**^**−04**^
Diabetes (self-reported)	361141	14114	43	0.92 (0.78, 1.08)	0.311	0.9 (0.82, 0.99)	**0.030**	0.85 (0.56, 1.29)	**2.48 × 10**^**−05**^
Diabetes (diagnosed)	360192	17275	55	0.99 (0.85, 1.15)	0.856	0.91 (0.83, 1)	0.052	0.86 (0.62, 1.21)	**3.51 × 10**^**−05**^
Malignant neoplasm of prostate	361194	6321	24	0.99 (0.85, 1.15)	0.891	0.88 (0.81, 0.95)	**6.72 × 10**^**−04**^	0.91 (0.29, 2.84)	**0.004**
Venous thromboembolism	361194	4620	10	0.95 (0.87, 1.04)	0.276	0.92 (0.87, 0.98)	**0.009**	0.92 (0.16, 5.36)	**0.015**
DVT of lower extremities	361194	2116	7	0.96 (0.9, 1.02)	0.201	0.95 (0.91, 0.99)	**0.024**	0.94 (0.06, 15.71)	**0.022**
DVT of lower extremities and pulmonary embolism	361194	4319	11	0.95 (0.87, 1.04)	0.239	0.93 (0.88, 0.99)	**0.017**	0.92 (0.17, 4.99)	**0.011**
No vascular/heart problems	360420	253565	170	1.36 (1.01, 1.82)	**0.045**	1.48 (1.31, 1.69)	**1.20 × 10**^**−09**^	1.55 (1.47, 1.64)	**3.98 × 10**^**−16**^
**Physical measures**									
Systolic blood pressure	340159	-	203	0.49 (0.25, 0.97)	**0.041**	0.54 (0.41, 0.71)	**1.00 × 10**^**−05**^	0.55 (0.44, 0.68)	**1.17 × 10**^**−07**^
Diastolic blood pressure	340162	-	178	0.77 (0.37, 1.59)	0.478	0.55 (0.42, 0.72)	**1.38 × 10**^**−05**^	0.56 (0.46, 0.69)	**1.20 × 10**^**−07**^
Arm fat mass (right)	354736	-	283	0.58 (0.39, 0.87)	**0.009**	0.6 (0.48, 0.74)	**4.47 × 10**^**−06**^	0.76 (0.66, 0.88)	**1.55 × 10**^**−04**^
Arm fat percentage (left)	354707	-	267	0.57 (0.3, 1.08)	0.087	0.53 (0.39, 0.72)	**4.83 × 10**^**−05**^	0.67 (0.54, 0.82)	**1.28 × 10**^**−04**^
Arm fat percentage (right)	354760	-	265	0.62 (0.32, 1.18)	0.144	0.52 (0.38, 0.71)	**3.85 × 10**^**−05**^	0.65 (0.53, 0.8)	**4.02 × 10**^**−05**^
Leg fat mass (right)	354807	-	287	0.46 (0.28, 0.78)	**0.004**	0.58 (0.44, 0.77)	**1.55 × 10**^**−04**^	0.67 (0.57, 0.8)	**5.95 × 10**^**−06**^
Leg fat mass (left)	354788	-	287	0.4 (0.24, 0.69)	**0.001**	0.54 (0.41, 0.72)	**2.60 × 10**^**−05**^	0.66 (0.55, 0.78)	**4.06 × 10**^**−06**^
Leg fat percentage (right)	354811	-	267	0.44 (0.13, 1.53)	0.196	0.42 (0.29, 0.61)	**7.54 × 10**^**−06**^	0.58 (0.42, 0.82)	**0.002**
Leg fat percentage (left)	354791	-	270	0.4 (0.12, 1.36)	0.142	0.4 (0.28, 0.58)	**1.46 × 10**^**−06**^	0.61 (0.44, 0.85)	**0.003**
Trunk fat mass	354597	-	298	0.67 (0.44, 1.03)	0.068	0.68 (0.55, 0.83)	**1.91 × 10**^**−04**^	0.77 (0.67, 0.89)	**2.71 × 10**^**−04**^
Whole body fat mass	354244	-	300	0.55 (0.37, 0.84)	**0.006**	0.61 (0.49, 0.76)	**9.41 × 10**^**−06**^	0.71 (0.62, 0.81)	**1.19 × 10**^**−06**^
Waist circumference	360564	-	252	0.73 (0.41, 1.32)	0.302	0.62 (0.46, 0.82)	**7.92 × 10**^**−04**^	0.73 (0.61, 0.88)	**0.001**
Body mass index: by impedance measurement	354831	-	260	0.64 (0.4, 1.04)	0.075	0.62 (0.49, 0.78)	**6.76 × 10**^**−05**^	0.73 (0.62, 0.86)	**1.70 × 10**^**−04**^
Body mass index: by height and weight measurement	359983	-	260	0.67 (0.42, 1.08)	0.104	0.63 (0.49, 0.79)	**8.47 × 10**^**−05**^	0.76 (0.65, 0.89)	**9.52 × 10**^**−04**^
**Family History**									
Father with AD/dementia	312666	15022	3	0.38 (0.13, 1.07)	0.068	0.39 (0.34, 0.44)	**2.59 × 10**^**−50**^	-	-
Mather with AD/dementia	331041	28507	5	0.41 (0.23, 0.72)	**0.002**	0.45 (0.39, 0.51)	**1.14 × 10**^**−33**^	0.46 (0.26, 0.81)	**5.88 × 10**^**−04**^
Father with heart disease	318570	104110	19	0.29 (0.11, 0.77)	**0.013**	0.49 (0.34, 0.71)	**1.46 × 10**^**−04**^	0.48 (0.29, 0.79)	**9.74 × 10**^**−04**^
Siblings with heart disease	280784	28696	3	0.33 (0.03, 4.21)	0.391	0.44 (0.28, 0.68)	**2.59 × 10**^**−04**^	-	-
Siblings with high blood pressure	281619	58495	15	0.37 (0.06, 2.38)	0.296	0.72 (0.48, 1.07)	0.101	0.57 (0.26, 1.23)	**0.006**
Siblings with none of group 1 disease*	281979	190969	5	34.04 (0.24, 4912.96)	0.164	3.3 (1.62, 6.73)	**9.92 × 10**^**−04**^	3.27 (0.89, 12.04)	**0.023**
**Medication**									
Aspirin	361141	48081	6	0.17 (0.04, 0.85)	**0.031**	0.34 (0.22, 0.52)	**9.03 × 10**^**−07**^	0.35 (0.15, 0.84)	**0.001**
Atorvastatin	361141	10805	18	0.48 (0.23, 0.99)	**0.048**	0.74 (0.65, 0.84)	**4.67 × 10**^**−06**^	0.64 (0.02, 18.99)	**0.006**
Amlodipine	361141	14588	18	0.63 (0.33, 1.22)	0.173	0.68 (0.57, 0.82)	**3.08 × 10**^**−05**^	0.71 (0.24, 2.06)	**3.17 × 10**^**−04**^
Metformin	361141	8968	31	0.94 (0.81, 1.09)	0.427	0.92 (0.84, 1.01)	0.092	0.89 (0.46, 1.74)	**5.03 × 10**^**−04**^
Simvastatin	361141	40921	33	0.72 (0.41, 1.27)	0.261	0.74 (0.6, 0.9)	**0.003**	0.65 (0.36, 1.15)	**2.89 × 10**^**−04**^
Bendroflumethiazide	361141	20196	32	0.46 (0.27, 0.8)	**0.006**	0.67 (0.58, 0.78)	**2.02 × 10**^**−07**^	0.7 (0.45, 1.09)	**1.00 × 10**^**−06**^
Blood pressure medication (females)	193148	33519	76	0.8 (0.59, 1.08)	0.148	0.74 (0.66, 0.83)	**1.84 × 10**^**−07**^	0.72 (0.66, 0.8)	**2.49 × 10**^**−10**^
Blood pressure medication (males)	165340	40987	56	0.67 (0.45, 1)	0.050	0.73 (0.63, 0.83)	**5.22 × 10**^**−06**^	0.69 (0.62, 0.77)	**1.30 × 10**^**−08**^
Cholesterol lowering medication (females)	193148	24247	28	0.77 (0.51, 1.15)	0.198	0.81 (0.7, 0.94)	**0.006**	0.74 (0.48, 1.14)	**9.27 × 10**^**−04**^
Cholesterol lowering medication (males)	165340	38057	38	0.69 (0.5, 0.95)	**0.024**	0.81 (0.69, 0.94)	**0.007**	0.74 (0.59, 0.91)	**1.54 × 10**^**−04**^
None of medication taken (females)	193148	133338	28	1.26 (0.48, 3.35)	0.641	1.72 (1.34, 2.21)	**1.94 × 10**^**−05**^	1.52 (1.1, 2.1)	**0.001**
None of medication taken (males)	165340	110372	49	1.43 (0.85, 2.42)	0.179	1.46 (1.23, 1.74)	**2.16 × 10**^**−05**^	1.53 (1.33, 1.76)	**1.56 × 10**^**−06**^
**Early life factors**									
Comparative height size at age 10	355331	-	225	0.69 (0.43, 1.1)	0.122	0.81 (0.63, 1.03)	0.088	0.77 (0.65, 0.92)	**0.004**
**Education**									
College or University degree	357549	115981	189	1.12 (0.72, 1.74)	0.616	1.29 (1.14, 1.47)	**9.95 × 10**^**−05**^	1.17 (1, 1.36)	**0.003**
**Lifestyle**									
Age first had sexual intercourse	317694	-	136	1.65 (0.52, 5.16)	0.394	1.74 (1.28, 2.38)	**4.59 × 10**^**−04**^	1.64 (1.32, 2.05)	**2.35 × 10**^**−05**^
**Diet**									
Never eat sugar or foods/drinks containing sugar	359777	67292	14	0.18 (0.02, 1.34)	0.093	0.62 (0.42, 0.91)	**0.016**	0.59 (0.31, 1.11)	**0.003**

*Group 1: heart disease, stroke, high blood pressure, chronic bronchitis/emphysema, Alzheimer's disease/dementia, and diabetes

*AD* Alzheimer’s disease, *COPD* chronic obstructive pulmonary diseases, *CI* confidence interval, *DVT* deep venous thrombosis, *MR-PRESSO* Mendelian randomization pleiotropy residual sum and outlier, *IVW* inverse variance weighted method, *OR* odds ratio, *SNP* single nucleotide polymorphisms

Among reported exposures in Fig. 2, forty-two traits were associated with both 90th and 99th percentile survival longevity outcomes. In the disease category, diseases of circulatory system (OR_90_ = 0.43 [0.32, 0.59], P_90_ = 1.0 × 10^−7^) were causally associated with lower odds of 90th and 99th percentile longevity. We observed that ischemic heart disease (OR_90_ = 0.66 [0.51, 0.87], P_90_ = 0.0029) was causally linked to both two longevity outcomes. MR-PRESSO global test and Q test showed substantial pleiotropy between the SNPs used as IVs for the two exposures (P < 0.05; Table 2). However, after removing potential outlying SNPs, the corrected MR-PRESSO results are still significant. For other heart disease-related traits, coronary atherosclerosis (OR_90_ = 0.77 [0.70, 0.84], P_90_ = 4.2 × 10^−8^), cardiac arrhythmias with chronic obstructive pulmonary diseases (OR_90_ = 0.86 [0.81, 0.92], P_90_ = 1.9 × 10^−5^), self-reported angina (OR_90_ = 0.72 [0.64, 0.82], P_90_ = 2.3 × 10^−7^), and diagnosed angina (OR_90_ = 0.73 [0.65, 0.83], P_90_ = 5.4 × 10^−5^) showed association with lower odds of 90th and 99th percentile longevity, while no vascular/heart problems (OR_90_ = 1.55 [1.41, 1.71], P_90_ = 1.9 × 10^−19^) showed the opposite. Pleiotropy tests were non-significant except for no vascular/heart problems (MR-Egger intercept P = 0.0002; global test P = 0.001). Nevertheless, the corrected MR-PRESSO result was still significant after removing outliers.

**Table 2:** Pleiotropy and heterogeneity analyses for the association between exposures and Alzheimer's disease in primary analysis

Exposome components	MR-Egger intercept P-value	Q test P-value	MR-PRESSO global test P-value	Corrected MR-PRESSO	
				OR (95%CI)	P-value
**Disease**					
Atrial fibrillation and flutter	0.87	0.82	0.89	-	-
Coronary atherosclerosis	0.11	0.08	0.16	-	-
Cardiac arrhythmias, COPD co-morbidities	0.81	0.33	0.80	-	-
Ischemic heart disease	1.66 × 10^−37^	0.46	< 0.001	0.74 (0.47, 1.15)	7.70 × 10^−06^
Angina (self-reported)	0.46	0.12	0.52	-	-
Angina (diagnosed)	0.54	0.07	0.55	-	-
Hypertension (self-reported)	1.11 × 10^−04^	0.32	< 0.001	-	-
Hypertension (diagnosed)	5.55 × 10^−05^	0.73	< 0.001	-	-
Diseases of the circulatory system	0.49	0.11	0.56	-	-
High cholesterol (self-reported)	1.92 × 10^−07^	0.32	< 0.001	0.79 (0.62, 1.01)	5.16 × 10^−05^
Diabetes (self-reported)	0.28	0.36	0.29	-	-
Diabetes (diagnosed)	0.24	0.07	0.25	-	-
Malignant neoplasm of prostate	0.35	0.29	0.35	-	-
Venous thromboembolism	0.19	0.35	0.31	-	-
DVT of lower extremities	0.48	0.34	0.53	-	-
DVT of lower extremities and pulmonary embolism	0.26	0.38	0.35	-	-
No vascular/heart problems	2.01 × 10^−04^	0.34	0.001	1.53 (1.45, 1.62)	1.43 × 10^−15^
**Physical measures**					
Systolic blood pressure	2.16 × 10^−08^	0.72	< 0.001	0.54 (0.44, 0.66)	3.10 × 10^−08^
Diastolic blood pressure	1.06 × 10^−04^	0.39	< 0.001	0.55 (0.45, 0.67)	1.39 × 10^−08^
Arm fat mass (right)	0.09	0.17	0.09	-	-
Arm fat percentage (left)	0.002	0.61	0.002	0.65 (0.53, 0.8)	4.99 × 10^−05^
Arm fat percentage (right)	0.005	0.87	0.005	0.64 (0.52, 0.78)	1.42 × 10^−05^
Leg fat mass (right)	0.11	0.14	0.13	-	-
Leg fat mass (left)	0.03	0.06	0.03	0.67 (0.57, 0.8)	8.71 × 10^−06^
Leg fat percentage (right)	2.94 × 10^−28^	0.64	< 0.001	0.51 (0.4, 0.65)	8.76 ×10^−08^
Leg fat percentage (left)	4.36 × 10^−23^	0.48	< 0.001	0.53 (0.43, 0.67)	1.15 × 10^−07^
Trunk fat mass	0.01	0.51	0.02	0.78 (0.68, 0.9)	4.58 × 10^−04^
Whole body fat mass	0.10	0.22	0.11	-	-
Waist circumference	2.90 × 10^−05^	0.99	< 0.001	0.7 (0.58, 0.84)	1.83 × 10^−04^
Body mass index: by impedance measurement	3.11 × 10^−09^	0.60	< 0.001	0.69 (0.6, 0.79)	8.48 × 10^−08^
Body mass index: by height and weight measurement	6.25 × 10^−08^	0.60	< 0.001	0.72 (0.63, 0.82)	1.06 × 10^−06^
**Family history**					
Father with AD/dementia	0.41	0.89	-	-	-
Mather with AD/dementia	0.18	0.15	0.40	-	-
Father with heart disease	0.006	0.24	0.01	0.5 (0.35, 0.72)	2.47 × 10^−04^
Siblings with heart disease	0.25	0.85	-	-	-
Siblings with high blood pressure	0.02	0.64	0.02	0.65 (0.3, 1.41)	0.016
Siblings with none of group 1 disease*	0.18	0.31	0.30	-	-
**Medication**					
Aspirin	0.46	0.24	0.59	-	-
Atorvastatin	2.15 × 10^−33^	0.36	< 0.001	0.72 (0.41, 1.26)	8.30 × 10^−07^
Amlodipine	0.04	0.72	0.05	-	-
Metformin	0.70	0.48	0.70	-	-
Simvastatin	3.75 × 10^−07^	0.67	< 0.001	0.63 (0.42, 0.93)	1.19 × 10^−05^
Bendroflumethiazide	0.18	0.11	0.21	-	-
Blood pressure medication (females)	0.02	0.50	0.03	-	-
Blood pressure medication (males)	0.04	0.86	0.04	-	-
Cholesterol lowering medication (females)	1.55 × 10^−06^	0.87	< 0.001	0.71 (0.56, 0.91)	9.32 × 10^−06^
Cholesterol lowering medication (males)	1.70 × 10^−05^	0.66	< 0.001	0.71 (0.62, 0.82)	3.07 × 10^−06^
None of medication taken (females)	0.010	0.70	0.01	1.62 (1.31, 2)	3.05 × 10^−05^
None of medication taken (males)	0.002	0.80	0.002	1.61 (1.43, 1.81)	4.23 × 10^−08^
**Early life factors**					
Comparative height size at age 10	0.004	0.62	0.006	0.78 (0.66, 0.93)	0.006
**Education**					
College or university degree	2.17 × 10^−04^	0.84	< 0.001	1.2 (1.07, 1.35)	2.03 × 10^−04^
**Lifestyle**					
Age first had sexual intercourse	0.18	1.00	0.19	-	-
**Diet**					
Never eat sugar or foods/drinks containing sugar	0.54	0.22	0.58	-	-

*Group 1: heart disease, stroke, high blood pressure, chronic bronchitis/emphysema, Alzheimer's disease/dementia, and diabetes

*AD* Alzheimer’s disease, *COPD* chronic obstructive pulmonary diseases, *CI* confidence interval, *DVT* deep venous thrombosis, *MR-PRESSO* Mendelian randomization pleiotropy residual sum and outlier, *IVW* inverse variance weighted method, *OR* odds ratio

Regarding blood pressure related traits, genetically predicted self-reported hypertension (OR_90_ = 0.68 [0.62, 0.74], P_90_ = 9.4 × 10^−17^) and diagnosed hypertension (OR_90_ = 0.70 [0.64, 0.77] P_90_ = 4.5 × 10^−14^) were significantly associated with higher odds of both two longevity outcomes, with no outlying genetic variant identified. Quantitative increase of systolic blood pressure (SBP; OR_90_ = 0.55 [0.44, 0.68], P_90_ = 3.9 × 10^−8^) and diastolic blood pressure (DBP; 0.56 [0.46, 0.69], P_90_ = 3.4 × 10^−8^) were also associated with higher odds of 90th and 99th percentile longevity. After removing potential outlying SNP through MR-PRESSO outlier test, significant effects remained of the two traits (Table 2).

We also noted an association between self-reported high cholesterol (OR_90_ = 0.81 [0.72, 0.91], P_90_ = 0.0003) and two longevity outcomes. After removing the outlying SNP identified by MR-PRESSO outlier test, the significant effects on 90th percentile longevity remained (Table 2). Besides, self-reported (OR_90_ = 0.85 [0.80, 0.91], P_90_ = 2.2 × 10^−6^) and diagnosed (OR_90_ = 0.86 [0.81, 0.92], P_90_ = 6.4 × 10^−6^) diabetes showed robust causal effects on both 90th and 99th percentile longevity without evidence of heterogeneity or pleiotropy. Malignant neoplasm of prostate (OR_90_ = 0.91 [0.86, 0.97], P_90_ = 0.0016) was also associated with lower odds of both two longevity outcomes without any evidence of heterogeneity or pleiotropy.

In the physical measures category, seven exposures referring to body morphology showed hazardous effects on both two longevity outcomes, including arm fat mass (right; OR_90_ = 0.76 [0.66, 0.88], P_90_ = 0.0001), arm fat percentage (right; OR_90_ = 0.65 [0.53, 0.80], P_90_ = 3.0 × 10^−5^), leg fat mass (right; OR_90_ = 0.67 [0.57, 0.80], P_90_ = 3.9 × 10^−6^), leg fat mass (left; OR_90_ = 0.66 [0.55, 0.78], P_90_ = 2.6 × 10^−6^), whole body fat mass (OR_90_ = 0.71 [0.62, 0.81], P_90_ = 7.1 × 10^−7^), waist circumference (OR_90_ = 0.73 [0.61, 0.88], P_90_ = 0.0012), and body mass index (BMI) measured by impedance measurement (OR_90_ = 0.73 [0.62, 0.86], P_90_ = 0.0001). Results of arm fat mass (right), leg fat mass (right), and whole body fat mass were robust for that no evidence of directional pleiotropy or heterogeneity were identified. Results of arm fat percentage (right), waist circumference, and BMI measured by impedance measurement showed potential pleiotropy, but they remain significant after removing outliers (Table 2). Remarkably, sitting height, standing height, weight, and body fat-free mass were unrelated with human longevity in a high power (see Additional file 1: Table S10 for more unassociated results).

Exposures of medication or family history were correlated with significant exposures of the disease category, including Alzheimer’s disease (AD) or dementia (father with AD/dementia: OR_90_ = 0.38 [0.34, 0.43], P_90_ = 6.0 × 10^−61^; mother with AD/dementia: OR_90_ = 0.46 [0.39, 0.54], P_90_ = 4.9 × 10^−23^), heart disease (father with heart disease: OR_90_ = 0.48 [0.33, 0.69], P_90_ = 8.4 × 10^−5^; siblings with heart disease: OR_90_ = 0.39 [0.26, 0.60], P_90_ = 1.7 × 10^−5^), hypertension (siblings with high blood pressure: OR_90_ = 0.57 [0.40, 0.81], P_90_ = 0.0014), and diabetes, chronic bronchitis/emphysema, stroke, and high cholesterol (siblings with none of these diseases: OR_90_ = 3.27 [1.71, 6.24], P_90_ = 0.0003). Regular taken of blood pressure medication, cholesterol lowering medication, metformin, and aspirin also showed significant association with lower odds of 90th and 99th percentile longevity. Besides, more medications taken (OR_90_ = 0.37 [0.18, 0.77], P_90_ = 0.008) was suggestively associated with lower odds of surviving to the 90th and 99th percentile age (see Additional file 1: Table S11-S12), while none medications taken showed the opposite (Fig, 2). In medication and family history category, results either showed no potential pleiotropy in MR-Egger intercept test or remained significant on 90th percentile longevity after removing outlying SNPs (Table 2).

Additionally, we found that comparative height size at age 10 (OR_90_ = 0.77 [0.65, 0.92], P_90_ = 0.0035) and never eat sugar or food/drinks containing sugar (OR_90_ = 0.59 [0.44, 0.80], P_90_ = 0.0005) showed association with lower odds of 90th and 99th percentile longevity. Substantial pleiotropy was only detected in comparative height size at age 10 (MR-Egger intercept P = 0.004; global test P = 0.006), but the result of corrected MR-PRESSO test was still significant after removing outlying variants.

Comparing results of 53 reported exposures in primary and in secondary analysis, four traits in disease category were only associated with 90th percentile survival longevity, including atrial fibrillation and flutter (OR_90_ = 0.91 [0.87, 0.96], P_90_ = 0.0002), deep venous thrombosis (DVT) of lower extremities (OR_90_ = 0.94 [0.91, 0.98], P_90_ = 0.0033), DVT of lower extremities and pulmonary embolism (OR_90_ = 0.92 [0.88, 0.97], P_90_ = 0.0018), and venous thromboembolism (VTE; OR_90_ = 0.92 [0.87, 0.97], P_90_ = 0.0028). Five body morphology traits in physical measures category were associated with lower odds of 90th percentile longevity, including arm fat percentage (left; OR_90_ = 0.67 [0.54, 0.82], P_90_ = 0.0001), leg fat percentage (right; OR_90_ = 0.58 [0.42, 0.82], P_90_ = 0.0017), leg fat percentage (left; OR_90_ = 0.61 [0.44, 0.85], P_90_ = 0.0032), trunk fat mass (OR_90_ = 0.77 [0.67, 0.89], P_90_ = 0.0002), and BMI measured by height and weight measurement (OR_90_ = 0.76 [0.65, 0.89], P_90_ = 0.0008). Besides, college or university degree (OR_90_ = 1.17 [1.06, 1.29], P_90_ = 0.0024) and age first had sexual intercourse (OR_90_ = 1.64 [1.32, 2.05], P_90_ = 1.2 × 10^−5^) were associated with higher odds of longevity only in 90th percentile data. All significant exposures identified in secondary analysis also showed significant results in primary stage.

### Validation

In the validation, the results of myocardial infarction (P_90_ = 0.036), coronary artery disease (P_90_ = 0.004), VTE (P_90_ = 0.017), AD (P_90_ = 3.0 × 10^−5^), trunk fat mass (P_90_ = 0.039), and education attainment (i.e., the number of years of schooling completed; P_90_ = 0.020) had secured our MR estimates in screening. Causal effects of LDL-C (OR_90_ = 0.72 [0.64, 0.80], P_90_ = 2.3 × 10^−9^), total cholesterol (OR_90_ = 0.71 [0.63, 0.81], P_90_ = 9.4 × 10^−8^), HDL-C (OR_90_ = 1.36 [1.13, 1.62], P_90_ = 0.001), triglycerides (OR_90_ = 0.82 [0.70, 0.96], P_90_ = 0.013), and type 2 diabetes (T2D; OR_90_ = 0.88 [0.80, 0.96], P_90_ = 0.004) were validated and supplemented our screening results of high cholesterol and diabetes. Atrial fibrillation (OR_90_ = 0.90 [0.80, 1.02], P_90_ = 0.089), prostate cancer (OR_90_ = 0.94 [0.88, 1.00], P_90_ = 0.063), type 1 diabetes (OR_90_ = 0.96 [0.81, 1.14], P_90_ = 0.642), BMI (OR_90_ = 0.97 [0.77, 1.23], P_90_ = 0.79), and waist circumference (OR_90_ = 0.85 [0.61, 1.18], P_90_ = 0.324) were well-powered but showed no causal effects on longevity (see Additional file 1: Table S10-S11). SBP and body fat mass were non-significant in the validation, but the statistical power to detect an effect was not enough to preclude the positive effects in primary analysis. Of all exposures in the validation, the Egger intercept test showed no pleiotropy.

After adjusting for genetic correlation between different types of blood lipids, the association between HDL-C and longevity was partially attenuated (OR_90_ = 1.29 [1.05, 1.60], P_90_ = 0.016). The hazardous effect of triglycerides was fully disappeared (OR_90_ = 0.99 [0.81, 1.21], P_90_ = 0.927), while that of LDL-C was still significant (OR_90_ = 0.68 [0.62, 0.76], P_90_ = 3.6 × 10^−13^).

### Potential components of human longevity exposome

**Fig. 4:**
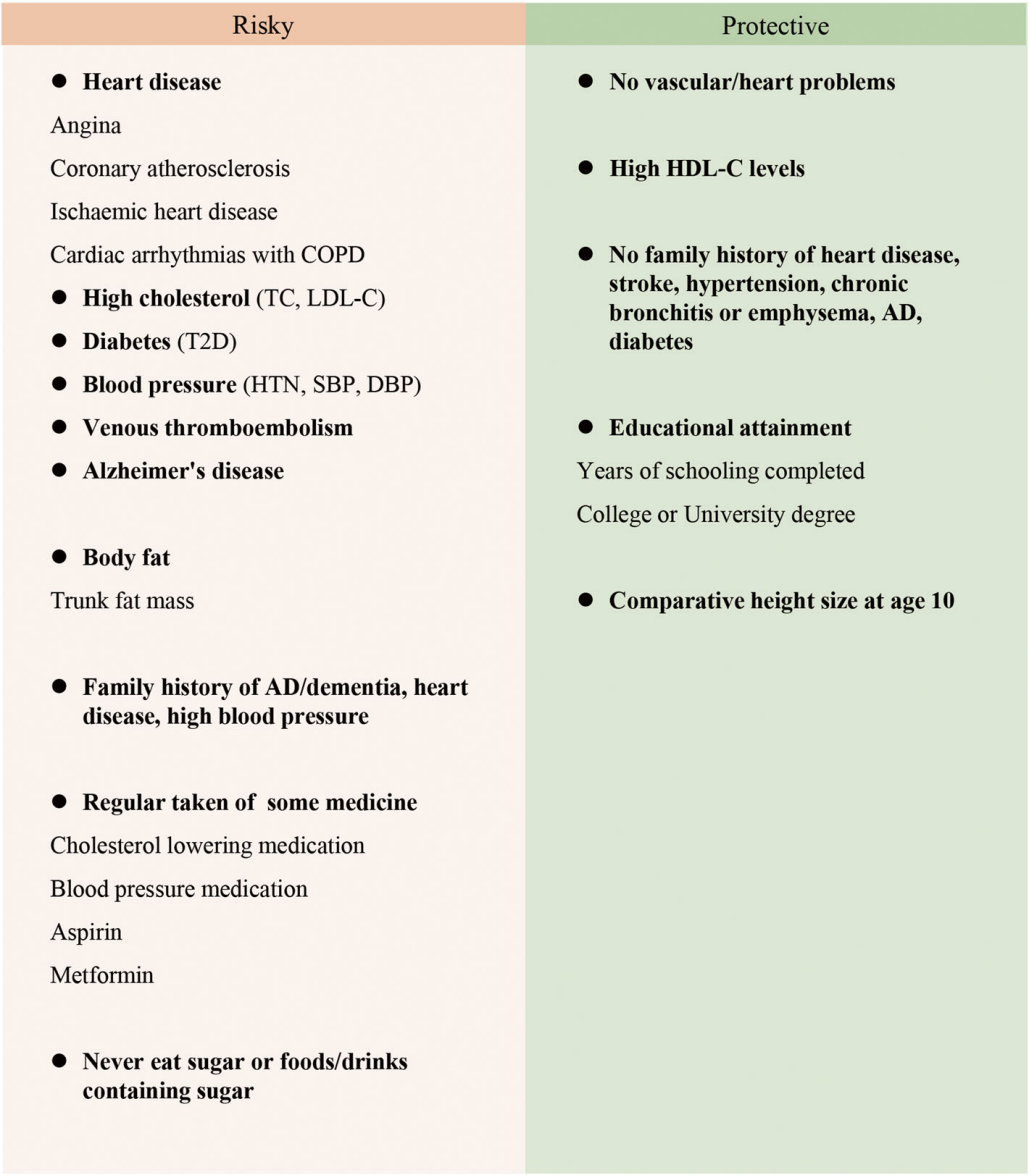
Components of the longevity exposome. COPD, chronic obstructive pulmonary diseases; DBP, diastolic blood pressure; HTN, hypertension; HDL-C, high-density lipoprotein cholesterol; LDL-C, low-density lipoprotein cholesterol; SBP, systolic blood pressure; T2D, type 2 diabetes; TC, total cholesterol

After screening and validation, robust exposures were considered components of longevity exposome, including 39 exposures that showed associations with both 90th and 99th percentile survival longevity at significant or suggestive levels in screening (see Additional file 1: Table S3), as well as VTE, AD, trunk fat mass, and educational attainment that were significant in the validation (Figs. 3 and 4). For note, malignant neoplasm of prostate, BMI, and waist circumference were excluded because of the non-significant validation results with a high power. Atrial fibrillation and flutter and age first had sexual intercourse were not considered components of longevity exposome for that the two results cannot be verified neither in secondary analysis nor in validation.

### Discussion

This is the first study using the MR approach to reveal causal components of longevity exposome. We found evidence that some heart diseases, metabolic syndromes, AD, VTE, greater body fat, higher comparative height size at 10, and never eat sugar or foods/drinks containing sugar have adverse effects on longevity, whereas higher HDL-C levels and higher education attainment have protective effects.

Our findings suggest the susceptibility to age-related diseases may significantly affect human longevity. Intuitively, our results have shown consistency with previous investigations. A progressive delay in the onset of age-related diseases, including ischemic heart disease, coronary atherosclerosis, angina, and AD, has been found with an association of increasing survival age [22]. Remarkably, GWAS have found that human longevity shared genetic correlations with CVD [3]. However, previous studies didn’t investigate the potential association using robust genetic analyses. By using MR method, we strengthen the potential causal effects of cardiovascular diseases on human longevity. Our MR study also demonstrated that hypertension, T2D, and higher LDL-C level were associated with lower odds of longevity, which is a strong confirmation of previous observational studies [2, 5, 8, 23]. It is believed to be causing genomic instability, telomere attrition, epigenetic alterations, and loss of proteostasis in the development of metabolic syndrome [24], thus leading to the reducing survival age. A healthy metabolic profile to avoid or delay the occurrence of metabolic syndrome may prolong longevity, as our results yield a positive association between age high blood pressure diagnosed and longevity at a suggestive level (see Additional file 1: Table S10-S12). Previous studies have also shown correlations between exceptionally healthy metabolic profile and human longevity [5, 25], shedding new insights for revealing the complexity of longevity. Furthermore, it is well known that many of those metabolic factors act as risk factors for CVD, metabolic syndrome, and AD. As these exposures of longevity interplay and intertwined, further studies are needed to decipher the pathways supporting these causal associations.

The protective effect of HDL-C was still significant in our study even after adjusting for LDL-C and triglycerides. As the genetically predicted HDL-C is not causally associated with CVD [26, 27], the relationship between HDL-C and longevity is unexpected and the underlying mechanism is not clear. HDL-C levels may affect longevity through complex relationships involving diverse factors [28]. Future studies focusing on the quality and components of HDL rather than the simple measurement of HDL may help to clarify the underlying mechanisms behind this relationship.

Despite some published studies have indicated an association between BMI and human lifespan [2], our results for BMI were conflicted among screening and validation stage. The conflicting results may be attributed to non-linear relation between BMI and longevity. As previous MR study and observational study showed, the relation between BMI and all-cause mortality is J-shaped [29, 30], and underweight is also correlated with higher risk of mortality. On the other hand, the relation of BMI and mortality is also affected by smoking status and age [29]. Thus, it is reasonable not to simply include higher BMI into the hazardous components of longevity exposome. However, some traits of body fat showed robust association with longevity, while body fat-free mass and weight were unrelated to longevity (see Additional file 1: Table S10). Based on these results, in terms of longevity, a practical recommendation is to reduce body fat than focus on the body fat-free mass or weight. Especially, higher trunk fat mass showed an association with human longevity. As a marker of central adiposity, it was linked with an increased risk of CVD and metabolic diseases [31], which may be one of the potential mechanisms.

Height in adulthood is believed to link with health and longevity, but the exact effect of height on human longevity is conflicted [32–35]. Our study clarified that standing height and sitting height were not associated with longevity at a suggestive level (see Additional file 1: Table S10-S12). However, higher comparative height size at 10 was negatively associated with human longevity. This result provided a different research prospective for investigation of relation between height and longevity.

Our results indicated a protective effect of higher education attainment, especially gaining college or university degree, on longevity. It is supported by previous evidences that higher life expectancies are associated with greater educational levels [36–38]. Education has also been proposed as a protective factor with both AD and CVD outcomes [39, 40]. Whether the protective effect of education on longevity is achieved by reducing the risk of CVD or AD needs further investigations.

Strengths of the study include the adoption of the MR approach for assessing the causal effects of a wide array of factors, getting the utmost out of large data and reducing selection bias. Our study identified some exposures that have never been investigated with MR frameworks of longevity, such as VTE, family history, body fat, diet, and comparative height in early life. Furthermore, the prudency on the definition of longevity phenotype has also allowed us to propose components of exposome causally linked to longevity more precisely since the definition of outcome was limited to mortality or parental life span in previous MR [2, 8]. Meanwhile, as with all MR studies, the exclusion of pleiotropy or alternative direct causal pathways is a conspicuous challenge. Although all the reported causal exposures in this study identified no pleiotropy in the Egger intercept test, significant Q-tests for some traits found substantial heterogeneity in the analysis. However, to avoid violation of MR assumptions, we conducted sensitivity analysis with weighted median, MR-Egger, and MR-PRESSO method. These methods can provide unbiased causal effect estimates at the cost of reduced power when invalid IVs exist [16, 17], and MR-PRESSO outlier test can return an unbiased result by removing potential outlying SNPs [18]. For each significant causal exposure in screening, the point estimates in sensitivity analyses were consistent with IVW, enhancing the robustness of our results [12]. Moreover, increased confidences were gained from the validation using independent exposure datasets. For exposures with vague phenotype descriptions in UKB, more detailed causal traits like LDL-C and T2D were included in the validation analysis using non-UKB exposure data.

There are some limitations to the present study. First, although we have used the largest data of longevity [3], the power of some exposures was below 80%. For example, smoking-related traits showed non-significant effects on longevity; however, because of the limited power (Additional file 2: Table S13-S14), we cannot preclude that they have effects on longevity. Second, not all significant exposures were able to conduct validation due to the lack of appropriate non-UKB data. It is important to note that the absence of a validation result does not disconfirm the robustness of a causal factor, but it also points to the need for further studies with a more comprehended exposure phenotype and a large sample size. Third, some of the exposures from UKB are ordinal variables but are treated as continuous when calculating betas for effect allele at each SNP, leading to difficulties in interpreting estimates quantitatively in subsequent MR analysis. In addition, the findings were discovered from participants of European ancestry that were recruited at the age between 40 and 69 that may not be generalizable to other populations [11]. Another limitation is that for some exposure GWASs, only sex stratified data were available in UK Biobank given that the outcome dataset is men and women combined. However, the effect estimates were very similar between men and women (Fig. 2), indicating the results were reliable. What is more, a few SNPs overlapped among some exposures, which may suggest that these exposures affect longevity by an interaction. Further studies are required to clarify the underpinning mechanisms of those causal associations.

Based on our findings, it is pellucid that the interventions on cardiovascular disease, metabolic syndrome, and AD, as well as VTE are in demand for the overall benefits of human longevity. Several preventions strategies have been proposed in published literatures and should be abundantly publicized [24, 39, 40]. We recommend people reducing body fat mass, especially the trunk fat mass, rather than simply focusing on losing weight. In the long term, receiving a higher-level education, at least gaining college or university degree, can generate persistent benefits for longevity. Moreover, appropriate intake of sugar or food/drinks containing sugar is recommended for the general population.

## Conclusions

In conclusion, by screening thousands of environmental factors for their association with human longevity in a MR framework, we proposed potential components of exposome that were causally linked to longevity. Our results supported the previous results that some age-related diseases, such as heart diseases, metabolic syndromes, and AD, are causally related to longevity. And we first reported the association between venous thromboembolism, never eat sugar or foods/drinks containing sugar, comparative height size at 10, and longevity. We also highlighted some powerful unrelated associations, such as sitting height, standing height, weight, and body fat-free mass. Prevention strategies should focus on modifying these risk factors and promote protective recommendations to improve longevity.

## Supplementary Information

**Additional file 1: Table S1.** Details of the instruments used as proxy risk factors for longevity. **Table S2.** Description of independent exposure GWAS used in validation. **Table S3.** Estimates of inverse variance weighted method for significant associations between genetically predicted exposures and longevity. **Table S4.** Sensitivity analysis results for the metabolites identified in secondary analysis. **Table S5.** Pleiotropy and heterogeneity analyses for the association between exposures and Alzheimer's disease in secondary analysis. **Table S6.** The Mendelian randomization association of individual SNPs of screening traits in primary analysis. **Table S7.** Mendelian randomization results of validation using independent exposure GWAS. **Table S8.** Mendelian randomization results of validation using dataset of 90th percentile longevity. **Table S9.** Mendelian randomization results of validation using dataset of 99th percentile longevity. **Table S10.** List of exposures in the present study. **Table S11.** Exposures identified significantly and suggestively associated with 90th percentile longevity in primary analyses. **Table S12.** Exposures identified significantly and suggestively associated with 99th percentile longevity in secondary analyses.

**Additional file 2: Table S13.** Mendelian randomization results of all traits in primary analysis using dataset of 90th percentile longevity. **Table S14**. Mendelian randomization results of all traits in secondary analysis using dataset of 99th percentile longevity.

## Data Availability

All the data used in this study can be acquired from the online data repository of Neale Lab, MR-Base, or from the individual referenced papers. Any other data generated in the analysis process can be requested from the corresponding author.

## Abbreviations

AD: Alzheimer’s disease
CVD: Cardiovascular disease
DBP: Diastolic blood pressure
FDR: False discovery rates
GWAS: Genome-wide association studies
HDL-C: High-density lipoprotein cholesterol
IV: Instrumental variables
IVW: Inverse variance weighted
LDL-C: Low-density lipoprotein cholesterol
MR: Mendelian randomization
MR-PRESSO: Mendelian randomization pleiotropy residual sum and outlier
OR: Odds ratios
SBP: Systolic blood pressure
SNP: Single nucleotide polymorphisms
T2D: Type 2 diabetes
VTE: Venous thromboembolism
